# Effects of Prolonged Exposure to Hypobaric Hypoxia on Oxidative Stress: Overwintering in Antarctic Concordia Station

**DOI:** 10.1155/2022/4430032

**Published:** 2022-04-30

**Authors:** Simona Mrakic-Sposta, Michela Montorsi, Simone Porcelli, Mauro Marzorati, Beth Healey, Cinzia Dellanoce, Alessandra Vezzoli

**Affiliations:** ^1^Institute of Clinical Physiology-Milan, National Research Council (CNR), Italy; ^2^Department of Human Sciences and Promotion of the Quality of Life, San Raffaele Roma Open University, Milan, Italy; ^3^Institute of Biomedical Technologies, National Research Council (CNR), Segrate, Milano, Italy; ^4^Department of Molecular Medicine, University of Pavia, Pavia, Italy; ^5^Biomedical Research, European Space Agency, Concordia, Canada

## Abstract

Concordia Station is the permanent, research station on the Antarctic Plateau at 3230 m. During the eleventh winter-over campaign (DC11-2015; February 2015 to November 2015) at Antarctic Concordia Station, 13 healthy team members were studied and blood samples were collected at six different time points: baseline measurements (T0), performed at sea level before the departure, and during the campaign at 3, 7, 20, 90, and 300 days after arrival at Concordia Station. Reducing the partial pressure of O_2_ as barometric pressure falls, hypobaric hypoxia (HH) triggers several physiological adaptations. Among the others, increased oxidative stress and enhanced generation of reactive oxygen/nitrogen species (ROS/RNS), resulting in severe oxidative damage, were observed, which can share potential physiopathological mechanisms associated with many diseases. This study characterized the extent and time-course changes after acute and chronic HH exposure, elucidating possible fundamental mechanisms of adaptation. ROS, oxidative stress biomarkers, nitric oxide, and proinflammatory cytokines significantly increased (range 24-135%) during acute and chronic hypoxia exposure (peak 20^th^ day) with a decrease in antioxidant capacity (peak 90^th^ day: -52%). Results suggest that the adaptive response of oxidative stress balance to HH requires a relatively long time, more than 300^th^ days, as all the observed variables do not return to the preexposition level. These findings may also be relevant to patients in whom oxygen availability is limited through disease (i.e., chronic heart and lung and/or kidney disease) and/or during long-duration space missions.

## 1. Introduction

Hypobaric hypoxia (HH), i.e., reduced partial pressure of oxygen, leads to decreased tissue oxygenation and a complex scenario of metabolic and physiological changes on an ascent to high altitude. Among the others, exposure to HH is associated with an increase in oxidative cellular damage due to the increased level of reactive oxygen (ROS) and nitrogen (RSN) species production and decreased antioxidant system activity [1–4].

Many lowlander individuals (skiers, trekkers, soldiers, astronomers, miners, guides, tourists, and porters) stay for long durations at high altitude due to occupational requirements and call of duty. Excessive ROS production is an indicator of oxidative stress (OxS), but it is also an integral part of a series of signalling events triggered by many stressful situations that can induce both an adaptive response or detrimental effects [5]. Indeed, the failure to adequately adapt to HH during chronic exposure to high altitude can result in hypoxemia and tissue oxidative damage inducing cellular apoptosis [6].

According to current understanding [2], increased ROS production associated with high altitude is due to different factors, including the mitochondrial respiratory chain, xanthine oxidase, and inducible nitric oxide synthase activity. ROS reactivity makes it challenging to measure, and the only and unique tool that allows direct ROS measurement is electron paramagnetic resonance (EPR) [7, 8]. The extent of OxS has been previously judged from the accumulation of the end-products of macromolecule interaction with ROS [9]. In particular, hypoxia-induced overproduction of ROS provokes oxidation of DNA, membrane lipids, and cell signalling proteins [10]. At high altitude, transient or sustained increase in many OxS-related biomarkers has been detected in plasma [1, 11].

As previously reported, at high altitudes, not only is there an increased amount of OxS but also it appears that the capacity of enzymatic and nonenzymatic antioxidant systems is somewhat decreased [1, 2]. Aminothiols are the major nonenzymatic antioxidant compounds that directly quench ROS. The most abundant is reduced glutathione (GSH), but various other aminothiols, including homocysteine (Hcy), cysteine (Cys), and cysteinylglycine (CysGly), are metabolically strictly related and can be considered the primary interface in the changing redox environment. Thiols are extremely efficient antioxidants that can protect cellular lipids, proteins, and nucleic acids against peroxidative damage due to their strong reductive capacity and ability to react with free radicals [12–14]. In plasma, aminothiols interact via redox and disulfide exchange reactions, generating a dynamic system referred to as redox thiol status that, by regulating cellular homeostasis, is a critical determinant of cell function [15].

Nitric oxide (NO) production plays a central role in conferring tolerance to hypoxia. Levett et al. [16] demonstrated that NO formation increases in lowlanders ascending to high altitude, in agreement with a role in adaptation to acute hypoxia. The release of NO bioactivity would facilitate hypoxic vasodilation in peripheral tissues and oppose hypoxic pulmonary vasoconstriction in the lungs [17]. A common and straightforward way of estimating body formation of NO, because of its extremely low levels and short half-life, is to measure its more stable oxidation products nitrate/nitrite in plasma as NO metabolites (NOx) that can be recycled back to bioactive NO, particularly in hypoxic conditions. Indeed, it has been suggested that nitrite anion (NO_2_^−^) may represent the major intravascular NO storage molecule whose reduction to NO is made possible through a nitrite reductase activity that is allosterically regulated with maximal activity at the hemoglobin P50 [18, 19].

Besides, blood plasma and/or red cells may stabilize and transport NO species that generate NO, particularly in hypoxic tissues. Attention has focused on the hypothesis that the allosteric transition, consequent to hemoglobin deoxygenation, may significantly link oxygen demand to the transport or generation of NO by hemoglobin [20]. It is generally accepted that the rapid reaction of NO with the heme groups of hemoglobin (Hb) produces a heme-iron nitrosyl adduct (Hb [FeNO]) but also sustains S-nitrosylation at Cys *β* 93 thiol residues. S-Nitrosohemoglobin (SNO-Hb) constitutes the primary conduit for circulating NO bioactivity and can be viewed as a principal regulator of SNO homeostasis, adaptively modulating NO chemistry to control NO bioactivity as a function of tissue oxygen saturation, thus serving as an O_2_ sensor and O_2_-dependent transducer of NO bioactivity [21, 22]. These studies raise the idea that NO bioactivity in vivo is dispensed to dilate blood vessels to the degree of hypoxia. Indeed SNO-Hb is very stable in the oxygenated structure and cannot effectively dilate blood vessels [22–24], but upon deoxygenation, the vasodilator potency is markedly potentiated since it behaves as a NO donor at low-oxygen tensions.

NO is also transported in blood and tissues as S-nitrosothiols (SNOs), which act as stable carriers and donors of NO and might signal tissue responses to hypoxia [22, 25].

It is known that hypoxia stimulates the expression of inflammatory cytokines [26] and increased ROS and oxidative stress are both recognized as critical proinflammatory mediators associated with inflammatory pathways [27].

Very few studies have been carried out on humans relating prolonged HH to the degree of oxidative stress [28, 29], inflammatory status [26], and NO production [16]. Moreover, controversies are reported regarding the timing of adaptations [30]. The source of such conflicting data may be related to interstudy differences in (1) intensity of exposure to hypoxia (rate of ascent and maximum height gained: ascent profile), (2) duration of exposure to hypoxia, (3) subjects' phenotypic characteristics, (4) exposure to other environmental stressors (e.g., cold, physical exertion, and altered energy balance), and (5) testing conditions (chamber, high altitude) [16].

Studies that investigate oxidative stress kinetic remodelling of responses to chronic hypoxia in a very controlled experimental condition are lacking.

Antarctica is a unique place to study health responses under an extreme environmental condition.

Regarding oxidative stress, only one study reports an increase in a marker of lipid peroxidation on 20 Ukrainians at the Ukrainian Antarctica station “Akademik Vernadsky” at sea level [31].

**Figure 1:**
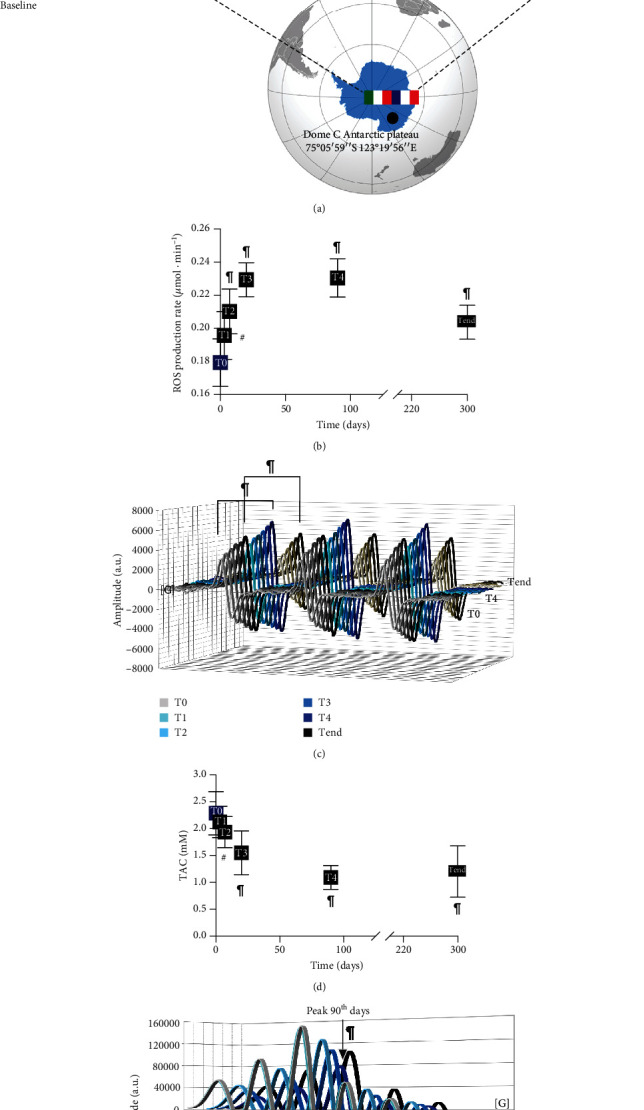
Experimental protocol and unbalance of ROS and TAC in acute and chronic hypobaric hypoxia. (a) Experimental timeline of the protocol adopted to monitor the acclimation effects during the winter-over campaign (DC11-2015) at Antarctic Concordia Station (637 hPa) on oxidative stress, aminothiol redox, inflammation status, NO metabolism, and Hb spectra. Monitored periods of study session from T0 (baseline) to Tend (300 days) are indicated. (b and d, respectively) The time course of ROS production rate (*μ*mol·min^−1^) and total antioxidant capacity (TAC) (mM) determined by EPR. (c) The stacked plots of the ROS EPR spectra recorded at baseline (T0), the 90^th^ day (T4), and the end (Tend) of sojourn at Concordia Station. (e) The stacked plots of the TAC EPR spectra recorded every time: from T0 to Tend.

For the first time, the present study evaluated the effects of acute and chronic hypobaric hypoxia in subjects exposed to high altitude in well-controlled experimental conditions on panels of oxidative stress/redox status and inflammatory biomarkers. Indeed, at Concordia Station in Antarctica, subjects are exposed to “moderate hypoxia” for up to 10 months (Figure 1(a)) without any change in altitude, thereby providing the opportunity to get an insight into the effects of moderate hypoxia in the absence of other disturbing factors. Concordia Station can provide fertile ground for biological and medical investigations for all these reasons. More specifically, we evaluated (1) ROS production and total antioxidant capacity (TAC), directly assessed by EPR; (2) oxidative damage and inflammation biomarker levels; (3) aminothiol redox status; (4) NO metabolism; and (5) UV-visible Hb spectra reflecting the time-related response to prolonged hypobaric hypoxia.

Findings from this work might contribute to understanding of the high-altitude acclimation/maladaptation mechanism. This study importance is due to the observation that oxidative stress damage is a potential physiopathological process associated with many disease states [7, 31, 32].

## 2. Materials and Methods

### 2.1. Concordia Research Station

The study was performed during the eleventh winter-over campaign (DC11-2015; February 2015 to November 2015) at Antarctic Concordia Station located on the high-ice plateau area called Dome C. Extreme climatic conditions and complete isolation (no access/exit possible) from the outer world during almost ten months (January to October) are the key characteristic features of this site. The temperature inside, where the team spent all of their time, was permanently 22 ± 2°C, and the mean barometric pressure (637 hPa or 478 mmHg) corresponded to an altitude of approximately 3233 m sl. However, accurate measurements have previously shown that the oxygen fraction in Antarctic air is lower than that in the rest of the world, and thus, it may be assumed that the actual equivalent altitude of Concordia is 3800 m sl [33].

### 2.2. Study Subjects

Thirteen healthy team members (10 males and 3 females) (mean age 34.1 ± 3.1 ys, BMI 24.8 ± 0.8 kg · m^−2^) volunteered to participate.

Under the Declaration of Helsinki, written informed consent was taken from all the subjects. The Commission for Research Bioethics of the Italian National Research Council (National Research Council, on 29/09/2014, decree nr. 0070015) and the Ethical Committee of the San Paolo Hospital in Milan (Prot. Gen on 05/05/2015 nr 5342) approved the study. All procedures and methods were performed following the relevant international guidelines and regulations to reduce the physical discomfort of the subjects. This study was conducted within the European Space Agency's (ESA) Life Science campaign at Concordia Station.

### 2.3. Blood Sampling

Blood samples were collected at six different time points. Baseline measurements (T0) were performed approximately two months before the departure to Antarctica at the European Space Agency headquarter in Cologne, Germany (91 m above sea level). During the campaign, blood venous samples were obtained 3, 7, 20, 90, and 300 days after arrival at Concordia Station. All samples were taken in the early morning and after an overnight fasting period. Strenuous physical exercise was not allowed for 24 h before sample collection. 5 mL of blood were drawn from the antecubital vein in heparinized vacuum tubes. The blood collected was centrifuged for 5 minutes at 3000 × *g* to separate plasma. Multiple aliquots were immediately frozen and stored at a minimum temperature of at least −40°C. At the end of the campaign, the frozen samples were transported to Italy by ship and plane in liquid nitrogen dewars with a controlled temperature (−80°C). After they arrived at Milan, plasma samples were stored at −80°C till further processing and analysis.

### 2.4. Hematological Parameters

Hematological parameters (i.e., total hemoglobin (Hb) and plasma level of erythropoietin (EPO)) and blood gas measurements were assessed. Some of us have previously reported detailed procedures and the assessed values for each parameter [33].

### 2.5. Electron Paramagnetic Resonance Measurements

Electron paramagnetic resonance (EPR) is a unique tool that allows direct measurements of free radical species [8, 34, 35]. Despite that, it is essential to note that no method for ROS detection is currently accepted as a “gold standard,” since every sensor or reagent has advantages and disadvantages including cyclic hydroxylamine spin-probes.

All EPR measurements were carried out on an X-band spectrometer (Escan, Bruker BioSpin, GmbH, MA, USA) equipped with a Temperature and Gas Controller “BIO-III” (Noxygen Science Transfer & Diagnostics GmbH, Germany) to assess the ROS production and total antioxidant capacity (TAC) at 37°C. Spectra acquired were recorded and analysed using Win EPR software (version 2.11) standardly supplied by Bruker.

For ROS production assessment, based on the method previously described [7] for each recruited subject, 50 *μ*L of plasma was treated with CMH (1-hydroxy-3-methoxycarbonyl-2,2,5,5 tetramethylpyrrolidine) probe solution (1 : 1). For data acquisition, 50 *μ*L of the obtained solution was put in a glass EPR capillary tube (Noxygen Science Transfer & Diagnostics, Germany) placed inside the cavity of the EPR spectrometer. All data were, in turn, converted in absolute concentration levels (*μ*mol·min^−1^) by adopting CP^•^ (3-carboxy-2,2,5,5 tetramethyl-1-pyrrolidinyloxy) stable radical as the external reference.

As previously reported, the antioxidant capacity was measured using 1,1-diphenyl-2-picrylhydrazyl (DPPH^•^) quenching [36]. Briefly, 5 *μ*L of plasma was added to 45 *μ*L of buffer solution (5 mM potassium phosphate (pH 7.4) containing 0.9% sodium chloride); then, the reaction was initiated by the addition of 50 *μ*L of DPPH^•^ 1 mM as a source of free radicals and incubated for 30 min at room temperature. Further, the sample was put in the glass EPR capillary tube (Noxygen Science Transfer & Diagnostics, Germany) placed inside the cavity of the EPR spectrometer for data acquisition. A linear calibration curve was computed from pure Trolox-containing reactions. The calculated antioxidant capacity was expressed in Trolox equivalent antioxidant capacity (TAC, mM).

### 2.6. Immune and Enzymatic Determinations of Plasma Oxidative Stress and Inflammation Biomarkers

All samples were assessed using a microplate reader spectrophotometer (InfiniteM200, Tecan, Austria). All the determinations were duplicated, and the interassay coefficient of variation was in the range indicated by the kit's manufacturer.

The malondialdehyde (MDA) levels were analysed spectrophotometrically using the modified thiobarbituric acid-reactive substance method to determine the amount of lipid peroxidation in plasma. The measurement of thiobarbituric acid-reactive substances (TBARS) by a commercial assay kit (Cayman Chemical, USA) allows a rapid photometric detection at 535 nm of the thiobarbituric acid malondialdehyde (TBAMDA) adduct, as previously reported [7]. A linear calibration curve was computed from pure MDA-containing reactions.

The protein carbonyl (PC) content, an index of protein oxidation, was determined utilizing a commercial kit (Cayman Chemical, USA) through the reaction of 2,4-dinitrophenylhydrazine (DNPH) and carbonyls. This reaction forms a Schiff base producing the correspondent hydrazone. The latter was analysed by spectrophotometry, reading the absorbance signal in the 360–385 nm range. Values were normalized to the total protein concentration in the final pellet (absorbance reading at 280 nm) to consider protein loss during the washing steps.

8-OH-2-deoxyguanosine (8-OH-dG), established as a marker of oxidative DNA damage, was assessed by using a commercially available enzyme immune assay EIA kit (Cayman Chemical, USA). The EIA employs an anti-mouse IgG-coated plate and a tracer consisting of an 8-OH-dG-enzyme conjugate, while the sample 8-OH-dG concentration was determined using an 8-OH-dG standard curve. Meanwhile, samples and standards were read at a wavelength of 412 nm.

Nitrite (NO_2_^−^)+nitrate (NO_3_^−^) (NOx) level determination was performed by the spectrophotometric method to Griess reagent, utilizing a commercial colorimetric assay kit (Cayman Chemical, USA).

Nitric oxide synthase (iNOS) expression was assessed by using a commercial assay EIA kit (cat no. EH0556; FineTest, Wuhan China). This assay was based on sandwich enzyme-linked immune-sorbent assay technology and carried out according to the manufacturer's instructions, while NOS2/iNOS protein synthesis was determined using a standard curve. Samples and standards were read at a wavelength of 450 nm.

Interleukin-6, interleukin-1*β*, and interleukin-10 (IL-6, IL-1*β*, and IL-10, respectively) levels were determined by using commercially available enzyme immune assay kits (R&D Systems, USA; Cayman Chemical, USA; and BioVendor, Czech Republic, respectively) following the manufacturer's instruction. The assays are based on a double-antibody sandwich technique. The signal was spectrophotometrically measured.

### 2.7. Thiol Determination

Oxidized (oxy) aminothiols were measured in the plasma according to previously validated methods [37]. The procedure adopted was the same utilized previously [7]. Briefly, Tris-(2carboxyethyl)-phosphine hydrochloride (TCEP) and 4-fluoro-7-sulfamoylbenzofurazan (ABD-F) were used as reducing and derivatizing agents, respectively; reduced aminothiols were assessed by mixing the plasma with 10% trichloroacetic acid (1 : 1 *v*/*v*). NaOH (0.4 M, 10 *μ*L), borate buffers (1 M, pH 11, 70 *μ*L as well as 1 M, pH 9.5, 30 *μ*L), each of them containing 4 mM EDTA, and ABD-F (10 g/L, 10 *μ*L, in borate buffer pH 9.5) were added to 100 *μ*L of the obtained supernatant. Samples were incubated at 4°C for 90 min, and then, 10 *μ*L was injected into the high-performance liquid chromatography (HPLC) system for analysis. Thiol separation was performed at room temperature by isocratic HPLC analysis on a Discovery C-18 column (250 × 4.6 mm I.D, Supelco, Sigma-Aldrich, St. Louis, MOS, USA), eluted with a solution of 0.1 M acetate buffer (pH 4.0) : methanol, 81 : 19 (*v*/*v*), at a flow rate of 1 mL·min^−1^. Fluorescence intensities were measured with an excitation wavelength at 390 nm and an emission wavelength at 510 nm, using a fluorescence spectrophotometer (Jasco, Japan). A standard calibration curve was used.

### 2.8. Hemoglobin UV-Visible Difference Spectra

Hb spectra were acquired using a computer-controlled microplate reader spectrophotometer (InfiniteM200, Tecan, Austria). The instrument recorded the absolute absorption spectrum of each plasma sample introduced into wells at 20°C remaining fixed throughout the experiment. Difference spectra were obtained by subtracting the UV-visible spectrum of plasma sample of each subject recorded at baseline from those recorded at 3, 7, 20, 90, and 300 days after arrival at Concordia Station. The difference spectra were generated arithmetically from the raw data.

### 2.9. Statistical Analysis

All data were expressed as mean ± SD. Data were analysed using parametric statistics following mathematical confirmation of a normal distribution using the Shapiro-Wilk test. Experimental data were compared by ANOVA and variance analysis followed by Bonferroni's multiple comparison test to further check among time groups' significance. A *p* < 0.05 value was considered statistically significant. Statistical analyses were carried out through the GraphPad Prism package (GraphPad Prism 9.0, GraphPad Software Inc., San Diego, CA, USA) and SPSS Statistic (IBM SPSS Statistic version 25, Hong Kong, China). Change Δ% estimation [((postvalue − prevalue)/prevalue)∗100] is also reported in the text.

## 3. Results

### 3.1. Acute and Chronic Hypobaric Hypoxia Induced High Levels of ROS Production and Decreased Antioxidant Capacity

It is well known that lowlanders switch on defence mechanisms when exposed to acute hypoxia, forming superoxide anion radicals and hydrogen peroxide. Few data are present about the effects of chronic hypoxia on ROS. This study showed that acute and chronic hypoxia exposure induced significant changes, from T0 to Tend, in the ROS production level, assessed by EPR. The ROS production rate (*μ*mol·min^−1^), recorded at any time, and the statistically significant differences between times of measurements are shown in Figures 1(b) and 1(c): T0 (0.17 ± 0.01) vs. T1 (0.19 ± 0.01), T2 (0.21 ± 0.01), T3 (0.23 ± 0.01), T4 (0.23 ± 0.01), and Tend (0.20 ± 0.01).

The ROS production increased immediately after three days of exposure, and it peaked after twenty days. Interestingly, the ROS production resulted elevated from T0 up to 10 months.

The redox status is mainly determined by the antioxidant capacity, which plays a key role in intracellular redox equilibrium and the metabolic regulation of the cellular defence against OxS [38]. The total antioxidant capacity in plasma and TAC concentration (mM), measured by EPR, at any time, showed statistically significant differences between times of measurements reported in Figures 1(d) and 1(e): T0 (2.29 ± 0.40) vs. T2 (1.93 ± 0.29), T3 (1.55 ± 0.41), T4 (1.09 ± 0.22), and Tend (1.20 ± 0.48). The decrease cooccurred simultaneously with the increase recorded in ROS production with a mirror kinetic.

### 3.2. Acute and Chronic Hypobaric Hypoxia Induced Changes of Oxidative Damage Biomarkers and Enhanced Availability of NO Metabolites

**Figure 2:**
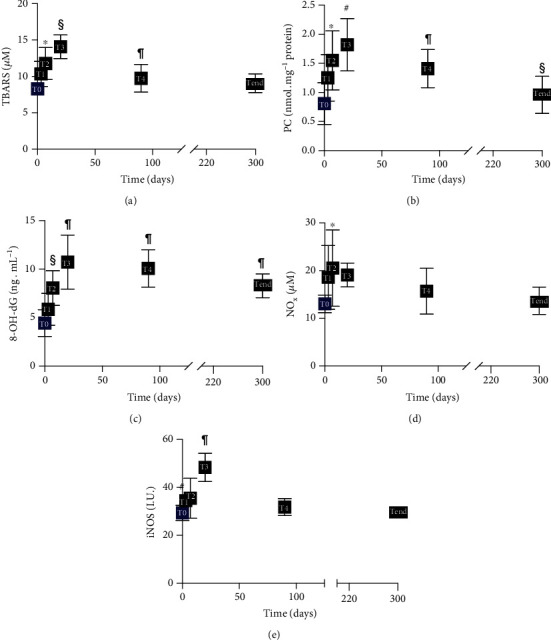
Acute and chronic hypobaric hypoxia-induced unbalance of oxidative stress and changes in nitric oxide metabolism: (a) thiobarbituric acid-reactive substances (TBARS) (*μ*M); (b) protein carbonyls (PC) (nmol·mg^−1^ protein); (c) 8-hydroxy-2-deoxyguanosine (8-OH-dG) (ng·mL^−1^); (d) nitric oxide metabolites (NOx) (*μ*M); (e) inducible nitric oxide synthase (iNOS) (I.U.), time course of concentration data. Data are shown as mean ± SD. Significant differences compared to T0 (baseline): *p* < 0.05 (∗ symbol).

The rise of ROS production was associated, at the same time, with an increase in oxidative damage to lipids (TBARS), proteins (PC), and DNA (8-OH-dG) (Figures 2(a)–2(c)). Their concentrations showed an increase after exposure and reached a peak after twenty days. More specifically, the differences between brackets in the figure were as follows: TBARS (*μ*M): T0 (8.29 ± 0.89) vs. T2 (11.77 ± 2.20), T3 (14.07 ± 1.64), and T4 (9.74 ± 1.90); PC (nmol·mg^−1^ protein): T0 (0.81 ± 0.36) vs. T2 (1.55 ± 0.51), T3 (1.82 ± 0.45), T4 (1.41 ± 0.33), and Tend (0.96 ± 0.32); and 8-OH-dG (ng·mL^−1^): T0 (4.44 ± 1.38) vs. T2 (8.06 ± 1.79), T3 (10.73 ± 2.79), T4 (10.08 ± 1.92), and Tend (8.28 ± 1.23).

Therefore, acclimatization to high altitude was accompanied by elevation of OxS and related oxidative damage. All these changes can have critical systemic implications [39].

Plasma NO_2_+NO_3_ (NOx) levels increased as reported in the literature [16], where NO formation increased in lowlanders ascending to high altitude, in agreement with a role in hypoxia adaptation. Results showed (Figure 2(d)) that after seven days, circulating NOx concentrations (*μ*M) resulted to be significantly increased: T0 (13.03 ± 1.87) vs. T2 (20.54 ± 8.00), after which a decrease to basal levels during acclimatization was observed. Moreover, iNOS levels (I.U.) significantly increased: T0 (29.35 ± 3.13) vs T3 (48.39 ± 5.92) (Figure 2(e)).

### 3.3. Redox Status Challenges during Hypobaric Hypoxia

Circulating total, reduced, and oxidized forms of aminothiols have been defined as “plasma redox status” [40]. The results (Table 1) demonstrated that exposure to HH is accompanied by markedly higher concentrations (*μ*mol·L^−1^) of oxidized Cys, CysGly, and GSH and total Hcy at twenty days. While oxidized Cys remained significantly higher during sojourn: T0 (193.93 ± 23.56) vs. T3 (251.83 ± 52.37), T4 (265.95 ± 57.57), and Tend (261.67 ± 56.04), oxidized CysGly and total Hcy tended to return to basal levels: CysGly T0 (26.30 ± 6.12) vs. T4 (33.58 ± 5.91) and Hcy T0 (8.41 ± 2.07) vs. T3 (12.26 ± 4.86). Oxidized GSH remained significantly higher at 90 days and then returned to the basal level during acclimatization: T0 (5.63 ± 1.81) vs. T3 (8.83 ± 2.29) and T4 (8.45 ± 1.40).

### 3.4. Cytokine Level Increased during Acute Hypobaric Hypoxia

**Figure 3:**
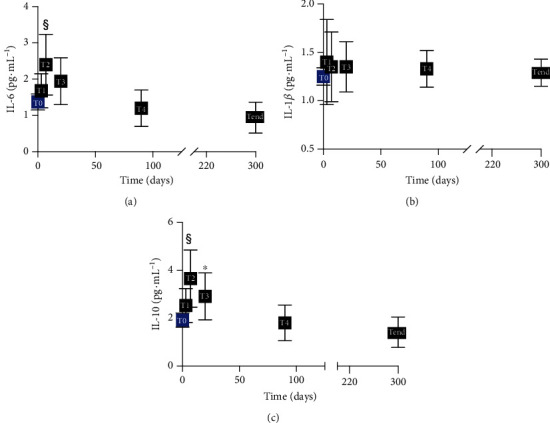
Inflammatory cytokines during acute and chronic HH. Time course of the IL-6, IL-1*β*, and IL-10 (pg·mL^−1^) in plasma. Data are shown as mean ± SD. Significant differences compared to T0 (baseline): *p* < 0.001 (§ symbol), *p* < 0.05 (∗ symbol).

Elevated levels of proinflammatory cytokines have been found in hypoxic subjects during ascent and sojourn to high altitude [26, 41]. A significant increase in IL-6 (pg·mL^−1^) after seven days at Concordia Station (T0 (1.37 ± 0.22) vs. T2 (2.40 ± 0.84)) was recorded (Figure 3(a)) compared to the sea level. Otherwise, no significant changes in IL-1*β* concentration were observed (Figure 3(b)).

Finally, a significant increase in IL-10 (pg·mL^−1^) at T2 (2.53 ± 0.71) and T3 (3.65 ± 1.20) was observed with respect to the sea level (1.91 ± 0.30) (Figure 3(c)).

### 3.5. Hb Reactions with Ligands Varied according to HH Acclimatization

The manifestation of hypoxia shows a decrease in HbO_2_ saturation and induces a series of physiological and pathological phenomena [42].

**Figure 4:**
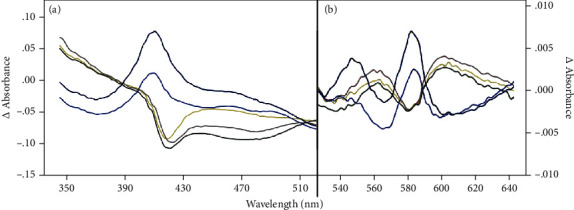
Difference Hb spectra. Representative absorption difference spectra (Δ absorbance) obtained from the data of spectra recorded every time subtracting the spectrum recorded at T0. The ocra yellow line: T1, light grey: T2, dark grey: T3, dark blue: T4, and black: Tend. *p* < 0.01 (# symbol), *p* < 0.001 (§ symbol), and *p* < 0.0001 (¶ symbol).

Typical kinetics of difference spectra recorded at different acclimatizing times are reported in Figure 4. A large decrease in the absorption value was observed at 422 nm (Figure 4(a)) and a similar variation but with an opposite sign at 555 and 596 nm (Figure 4(b)) in 3-, 7-, and 20-day plasma sample difference spectra. Otherwise, in 90- and 300-day difference spectra, increases in the absorption value were observed at 414 and 577 nm. Wavelength absorptions of 414 and 577 nm correspond to human HbO_2_ and 555 to deoxHb [43]. Otherwise, the absorptions at 422 and 596 nm correlated with iron nitrosyl Hb [44–46]. The net result is a change from oxy to deoxy Hb during the first phase of acclimation, followed by an increase in Hb concentration [33] to support the O_2_ supply.

### 3.6. Hematological Parameter Variations

The change percentages at T1, T2, T3, T4, and Tend with respect to the baseline (T0) of total Hb (g/dL) and EPO (mIU/mL) previously reported [33] and change percentage of Emogas values (pH, pO_2_, pCO_2_ mmHg, SaO_2_%, and base excess mEq/L) at 150 and 300 days with respect to T0 also reported by some of us [33] are shown in Table 2.

## 4. Discussion

The novelty of the current study was to investigate the effects of long-term exposure to hypobaric hypoxia, in the absence of poorly controllable disturbing stressors, on oxidative stress and inflammatory and redox status determined at the plasma level in humans. Chronic exposure to moderate hypoxia showed a modification in the oxidant/antioxidant balance through a great OxS response and transient but significant changes in the inflammatory responses.

The reported data help to provide accurate kinetics of the adaptations, understanding the effects of chronic hypobaric hypoxia on humans. Exposure to moderate or high altitude can determine potential medical problems when hypobaric hypoxia exposure is accompanied by a lack of adaptation, as changes in OxS balance may highlight. Our findings may be relevant not only for the subjects exposed to hypobaric hypoxia but also for the patients in limited oxygen delivery and/or affected by heart, vascular, and lung diseases.

Oxidative stress can be due to the increased ROS production level and/or the decreased level of antioxidant capacity. This study showed that chronic exposure to hypobaric hypoxia perturbed oxidative stress balance by an increased ROS production that induces severe oxidative damage from one side and a reduction of the TAC and redox thiol system from the other (Figure 1 and Table 1). Although an increased generation of ROS may be an essential stimulus to initiate adaptive responses to hypoxia, according to Semenza [47] theory of a common sensing mechanism for both hypoxia and ROS, inadequate antioxidant defences in the face of increased oxidative stress could lead to maladaptation and culminate into high-altitude-related pathologies: metabolic, hemodynamic, and clinical (i.e., heart failure, pulmonary disease, and cognitive function) diseases [39, 48, 49].

Our study showed that the balance between antioxidant capacity and oxidants was impaired shortly after arrival at Concordia Station, and this effect was never completely reversed. Thus, the subjects could only be able to partially adapt to hypobaric hypoxia even after 300 days. Moreover, for the first time, these data showed that OxS does not adapt to hypoxia, and they also confirmed the results presented by our group on the same subjects about blood gas data analysis [33].

Even if the acclimatization to each individual's hypoxia response seems to be affected by distinct differences, a significant increased ROS production and elevated oxidative damage to lipids, proteins, and DNA have been consistently reported [2, 39]. In this study, protein oxidation, lipid peroxidation, and DNA damage significantly increased in the first days of hypoxic exposure, reaching a maximum at 20 days of high-altitude sojourn according to the higher ROS production rate and the decrease in antioxidant defence during the initial months of acclimatization. However, during the following months at high altitude, PC, TBARS, and 8-OH-dG levels declined to indicate the activation of an adaptive response attempting to cope with free-radical formation resulting anyway insufficient as OxS biomarker levels remained still significantly higher than preexposure. This observation supports the view that induction of oxidative stress might be a trigger event in the acclimatization process.

As reported previously [29], long-term acclimatization and/or genetic adaptation attenuate or eliminate the high altitude-induced oxidative stress. From the blood samples of soldiers of an army unit transferred to high-altitude areas after 3 and 13 months of exposure, it appeared that 3 months is an adequate time frame to cause increased lipid peroxidation and decreased enzymatic along with nonenzymatic defences, while a 13-month sojourn normalizes the redox balance.

Low TAC levels could be due to increased utilization during neutralization of free radicals and inadequate activation of other antioxidant defence mechanisms in the body.

Aminothiols provide the first line of defence against oxidative stress by acting as a trap for ROS generated in aqueous compartments such as plasma, cytosol, and other body fluids. As aminothiol defence machinery is organized as a network and operates in an integrated manner, every component of the network can affect the functioning of another part to regenerate reduced glutathione, the effective primary scavenger of free radicals. Higher levels of recorded Cys and CysGly oxidized forms confirm this mechanism (Table 1).

However, data of this study, even if pointed toward a resumption of homeostatic balance between the generation of free radicals and antioxidant defence, show up that 10 months is not sufficient for a completed restoration.

Himalayan natives demonstrated adaptation to oxidative stress by developing specific intrinsic mechanisms protecting them from oxidants [50]. These altitude natives, particularly Sherpas, might have activated an enzymatic detoxifying ROS system more efficiently than acclimatized lowlanders, and higher enzyme activity may have a genetic basis.

Studies by Ashraf et al. [51] have revealed that high-altitude sojourn could result in hyperhomocysteinemia, a risk factor for arterial and venous thrombosis, and is accepted as an independent predictor of cardiovascular diseases [52]. The binding of nitric oxide with vitamin B12 and its precursors, resulting in inhibition of methionine synthase activity, could be a plausible reason for increased homocysteine concentration as observed in previous studies [53, 54]. A similar increase was recorded in the present study in the initial exposition phase (<20 days) and returned to baseline levels.

The hypobaric hypoxia response represents a complex network of biological pathways in which the nitrergic system plays an important role [55]. NO generated by the inducible form of NO synthase (iNOS) has been implicated in physiopathological states, and the NO production can exhibit a double effect: positive, if attributed to a decrease in neutrophil/platelet adhesion or vasodilation, or a negative, if related to the production of free radicals [56]. An increase in NO concentration following high-altitude exposure has been reported [57, 58]. Given the significance of NO in the regulation of so many vital bodily functions, including vascular tone and mitochondrial activity, enhanced availability of NO would seem to be essential for sustained local NO signalling under conditions of globally elevated ROS production, as we here documented to occur on a sojourn to high altitude. The enzymatic formation of NO from L-arginine by nitric oxide synthases is an oxygen-requiring process and, therefore, probably inhibited in our experimental conditions. On the contrary, the production of NO may come through serial reduction of inorganic nitrate (NO_3_^−^) to nitrite (NO_2_^−^), and NO is generally inhibited by oxygen [58]. Our observation of increased NOx levels in plasma above baseline by 7 days of exposure to a high altitude not the sustained on prolonged stay was reported previously [13]. Therefore, transient NOx accumulation is an early but unstained response [59]. On the other hand, iNOS increase in our subject among 20 days of hypobaric exposure.

Redox state influences the equilibria between thiols/nitrosothiols that convey NO bioactivity, and a release of NO by redox activation of plasmatic storage forms was reported by the drop in S-nitroso species too [13]. The increase in total thiol levels observed (Table 1) might confirm the release.

In response to tissue hypoxia, local vasodilation is a fundamental physiologic response that ensures oxygen delivery to respiring tissues under metabolic stress [60]. Hemoglobin acting as the hypoxic sensor that couples decreasing oxygen tension to increased blood flow contributes to vasodilation in response to hypoxia. Proposed mechanisms include S-nitrosohemoglobin- (SNO-Hb-) dependent vasodilation [24, 61]. Upon deoxygenation (see an increase in 555 nm absorbance values in Figure 4(b)), nitric oxide may be released from Cys93 and captured by a vacant heme to reform HbNO (Figures 4(a) and 4(b)). A small amount of the NO “escapes” this autocapture to elicit vasodilation in response to demand [62*].* When Hb concentration increased (see the increase in 414 and 577 nm absorbance values in Figures 4(a) and 4(b)) as previously reported too [33], HbNO decreased probably with the formation of HbSNO by the transfer of NO to the thiol group [21, 63] and/or from the reaction between deoxy-Hb and nitrite [62*].*

Cytokine release may mediate physiological adaptations to stress, and hypoxia functions as a danger signal for the immune system by inducing the synthesis of inflammatory cytokines. Indeed, the responses to low oxygen concentration have evolved as a physiological mechanism to detect tissue injury and improve tissue repair. However, these responses are potentially harmful at high altitudes too. Several studies examined cytokines at high altitudes. Data of the present study confirm those of Hartmann et al. that reported increased IL-6 levels in healthy volunteers who spent 3 nights at an elevation above 3400 m [26]. The IL-6 level changes (Figure 3) coincided temporally with the increases in ROS production. IL-6 is a reliable and sensitive marker of systemic inflammation, but the level recorded in the present study was below the range expected for inflammatory diseases. It significantly increased only in the acute phase (<7 days); therefore, the oxi-inflammatory biomarker IL-6 may not represent a stress mediator of chronic exposure to hypoxia. Moreover, our data confirm in vivo that IL-1*β* secretion was not induced at altitude [64]. To conclude, to support our finding in IL-10 changes after 20 days, murine studies exposed to short-term hypoxia have shown higher plasma concentrations of circulating IL-10 [65] and other inflammatory cytokine response. Furthermore, Dziurla et al. [66] in cell culture exposed in vitro to hypoxia found increased IL-10 and IL-8.

In contrast to most of proinflammatory cytokines, IL-10 mainly exerts anti-inflammatory effects, and the crucial task of IL-10 is the inhibition of the acute inflammation response [67]. Therefore, we can deduce that the increase in IL-10 in the first 20 days of hypobaric hypoxia leads to a form of acclimatization.

## 5. Limitation

The small number, for logistical reasons, of subjects severely limited statistical power, and there was no control group. Moreover, there was no chance of calling back the participants in order to monitor the follow-up at the end of the expedition, as they were of various nationalities.

However, the power and the novelty of this longitudinal study design consist of evaluating ROS production and many biomarkers of oxidative status in both the acute and chronic phases of hypobaric hypoxia exposure in Antarctic Concordia Station thus in the absence of poorly controllable disturbing stressors.

## 6. Conclusion

Exposure to high altitude increases ROS production, disrupting the efficiency of the antioxidant system and leading to oxidative damage to macromolecules. The present study results suggest that humans display little capacity for hypoxia acclimatization even after ten months of constant exposure to reduced PO_2_ without confounding factors. Moreover, antioxidant upregulation may play an essential role in adapting hypoxia-mediated oxidative stress at high altitudes. Hence, strengthening antioxidant defence by an exogenous supply of antioxidants may probably quicken acclimatization to oxidative stress as resulted from preliminary reports [68].

Finally, we think that our results, especially if correlated in other future researches with physiological parameters (i.e., measures of blood flow, ventilation, gas exchange in the lungs, endothelial function, and cardiovascular echoimaging) could be helpful in developing strategies to find appropriate and preservative solutions for human health and well-being in extreme/environmental conditions, such as space travels.

## Figures and Tables

**Table 1:** Thiol kinetics during acute and chronic hypobaric hypoxia. Time course of the total, reduced, and oxidized aminothiol concentrations (*μ*mol·L^−1^) in plasma. Data are shown as mean ± SD. Significant differences compared to T0 (baseline): *p* < 0.05 (∗ symbol), *p* < 0.01 (# symbol), and *p* < 0.001 (§ symbol). Cys: cysteine; CysGly: cysteinylglycine; Hcy: homocysteine; GSH: glutathione.

Plasma	T0	T1	T2	T3	T4	Tend
Cys						
Total	200.95 ± 24.41	228.37 ± 40.38	234.44 ± 40.78	259.39 ± 53.94^∗^	274.95 ± 59.62^#^	269.33 ± 57.68^∗^
Reduced	7.02 ± 0.85	6.53 ± 1.00	6.72 ± 1.05	7.55 ± 1.57^∗^	8.99 ± 2.06	7.67 ± 1.69
Oxidized	193.93 ± 23.56	221.84 ± 39.42	227.72 ± 39.74	251.83 ± 52.37^∗^	265.95 ± 57.57^#^	261.67 ± 56.04^∗^
Hcy						
Total	8.41 ± 2.07	11.11 ± 2.76	11.95 ± 2.90	12.26 ± 4.86^∗^	9.83 ± 2.56	8.53 ± 2.30
CysGly						
Total	28.59 ± 6.01	30.59 ± 5.94	31.71 ± 5.46	33.60 ± 6.75	35.97 ± 6.02^∗^	32.80 ± 6.01
Reduced	2.34 ± 0.32	2.71 ± 0.31	2.80 ± 0.70	3.21 ± 0.77^§^	2.39 ± 0.32	2.25 ± 0.33
Oxidized	26.30 ± 6.12	27.91 ± 5.80	28.91 ± 5.67	30.39 ± 6.94	33.58 ± 5.91^∗^	30.55 ± 6.24
GSH						
Total	6.27 ± 1.89	7.13 ± 2.03	8.22 ± 2.11	9.89 ± 2.25^§^	9.36 ± 1.60^#^	7.55 ± 1.44
Reduced	0.64 ± 0.19	0.76 ± 0.38	0.80 ± 0.42	1.06 ± 0.17^∗^	0.92 ± 0.39	0.58 ± 0.33
Oxidized	5.63 ± 1.81	6.40 ± 1.86	7.40 ± 2.28	8.83 ± 2.29^§^	8.45 ± 1.40^#^	6.97 ± 1.27

**Table 2:** Hematological and blood gas measurements. Change % of hematological (total Hb and EPO) measurements in subjects at T1, T2, T3, T4, and Tend with respect to T0 and blood gas measurements (pH, pO_2_, pCO_2_ mmHg, SaO_2_%, and base excess) at 150 and 300 days with respect to T0.

Hematological measurements					
	%T1	%T2	%T3	%T4	%Tend
Total Hb (g/dL)	+3	+12.5	+15	+20.5	+15
EPO (mIU/mL)	+193	+72	+26	-3.5	-6
Capillary blood gas measurements					
				% 150 days	%Tend
pH				+0.9	+1
pO_2_				-2	-0.6
pCO_2_ mmHg				-41	-40.5
SaO_2_ (%)				-0.8	-0.3
Base excess mEq/L				-246	-204

## Data Availability

All data associated with this study are present in the paper. All data generated in this study are available from the authors upon reasonable request.

## Abbreviations

BMI: Body mass index
Cys: Cysteine
CysGly: Cysteinylglycine
CMH: 1-Hydroxy-3-methoxycarbonyl-2,2,5,5 tetramethylpyrrolidine
CP^•^: 3-Carboxy-2,2,5,5 tetramethyl-1-pyrrolidinyloxy
DPPH^•^: 1,1-Diphenyl-2-picrylhydrazyl
EPR: Electron paramagnetic resonance
GSH: Glutathione
Hb: Hemoglobin
Hb [FeNO]: Heme-iron nitrosyl
HH: Hypobaric hypoxia
HPLC: High-performance liquid chromatography
Hcy: Homocysteine
IL-1*β*, IL-6: Interleukin-1*β*, interleukin-6
NO: Nitric oxide
NO_2_^−^: Nitrite anion
NO_3_^−^: Nitrate anion
iNOS: Inducible nitric oxide synthase
OxS: Oxidative stress
PC: Protein carbonyl
SNO-Hb: S-Nitrosohemoglobin
RNS: Reactive nitrogen species
ROS: Reactive oxygen species
TAC: Total antioxidant capacity
TBARS: Thiobarbituric acid-reactive substances
8-OH-dG: 8-OH-2-deoxyguanosine.

## Additional Points

*Highlights.* (i) Time-course changes in acute and chronic (300 days) hypobaric hypoxia were monitored. (ii) Kinetics of ROS and TAC levels were assessed by EPR during 300 days in Antarctica. (iii) Adaptive response of OxS to HH exposure requires a relatively long time. (iv) In acute hypoxia, NOx, iNOS, and IL-6 were significantly higher at altitude than at sea level. (v) Confounding factors such as lifestyle and dietary and physical activity were controlled.
